# Myeloid *miR-155* plays a limited role in antibacterial defense during *Klebsiella*-derived pneumosepsis and is dispensable for lipopolysaccharide- or *Klebsiella*-induced inflammation in mice

**DOI:** 10.1093/femspd/ftad031

**Published:** 2023-10-19

**Authors:** Wanhai Qin, Anno Saris, Cornelis van ’t Veer, Joris J T H Roelofs, Brendon P Scicluna, Alex F de Vos, Tom van der Poll

**Affiliations:** Center for Experimental and Molecular Medicine, Amsterdam University Medical Centers, Academic Medical Center, University of Amsterdam, 1105 AZ Amsterdam, The Netherlands; Amsterdam Infection and Immunity Institute, 1105 AZ Amsterdam, The Netherlands; Center for Experimental and Molecular Medicine, Amsterdam University Medical Centers, Academic Medical Center, University of Amsterdam, 1105 AZ Amsterdam, The Netherlands; Amsterdam Infection and Immunity Institute, 1105 AZ Amsterdam, The Netherlands; Center for Experimental and Molecular Medicine, Amsterdam University Medical Centers, Academic Medical Center, University of Amsterdam, 1105 AZ Amsterdam, The Netherlands; Amsterdam Infection and Immunity Institute, 1105 AZ Amsterdam, The Netherlands; Department of Pathology, Amsterdam University Medical Centers, Academic Medical Center, University of Amsterdam, 1105 AZ Amsterdam, The Netherlands; Amsterdam Cardiovascular Sciences, University of Amsterdam, 1105 AZ Amsterdam, The Netherlands; Center for Experimental and Molecular Medicine, Amsterdam University Medical Centers, Academic Medical Center, University of Amsterdam, 1105 AZ Amsterdam, The Netherlands; Amsterdam Infection and Immunity Institute, 1105 AZ Amsterdam, The Netherlands; Department of Applied Biomedical Science, Faculty of Health Sciences, Mater Dei Hospital, University of Malta, MSD 2080, Msida, Malta; Centre for Molecular Medicine and Biobanking, University of Malta, MSD 2080, Msida, Malta; Center for Experimental and Molecular Medicine, Amsterdam University Medical Centers, Academic Medical Center, University of Amsterdam, 1105 AZ Amsterdam, The Netherlands; Amsterdam Infection and Immunity Institute, 1105 AZ Amsterdam, The Netherlands; Center for Experimental and Molecular Medicine, Amsterdam University Medical Centers, Academic Medical Center, University of Amsterdam, 1105 AZ Amsterdam, The Netherlands; Amsterdam Infection and Immunity Institute, 1105 AZ Amsterdam, The Netherlands; Division of Infectious Diseases, Amsterdam University Medical Centers, University of Amsterdam, 1105 AZ Amsterdam, The Netherlands

**Keywords:** miR-155, myeloid cells, inflammatory responses, anti-bacterial defense, lung inflammation, pneumosepsis

## Abstract

MicroRNA-155 (*miR-155*) plays a crucial role in regulating host inflammatory responses during bacterial infection. Previous studies have shown that constitutive *miR-155* deficiency alleviates inflammation while having varying effects in different bacterial infection models. However, whether *miR-155* in myeloid cells is involved in the regulation of inflammatory and antibacterial responses is largely elusive. Mice with myeloid cell specific *miR-155* deficiency were generated to study the *in vitro* response of bone marrow-derived macrophages (BMDMs), alveolar macrophages (AMs) and peritoneal macrophages (PMs) to lipopolysaccharide (LPS), and the *in vivo* response after intranasal or intraperitoneal challenge with LPS or infection with *Klebsiella (K.) pneumoniae* via the airways. *MiR-155-*deficient macrophages released less inflammatory cytokines than control macrophages upon stimulation with LPS *in vitro*. However, the *in vivo* inflammatory cytokine response to LPS or *K. pneumoniae* was not affected by myeloid *miR-155* deficiency. Moreover, bacterial outgrowth in the lungs was not altered in myeloid *miR-155*-deficient mice, but *Klebsiella* loads in the liver of these mice were significantly higher than in control mice. These data argue against a major role for myeloid *miR-155* in host inflammatory responses during LPS-induced inflammation and *K. pneumoniae*-induced pneumosepsis but suggest that myeloid *miR-155* contributes to host defense against *Klebsiella* infection in the liver.

## Teaser

The study argues against a major role for myeloid miR-155 in host inflammatory responses during LPS-induced inflammation and Klebsiella pneumoniae-induced pneumosepsis but suggests that myeloid miR-155 contributes to constraining Klebsiella infection.

## Introduction

Macrophage responses during bacterial infections and inflammatory conditions are regulated at multiple levels (Zhang and Cao 2019, 2021). *MiR-155* is an important noncoding RNA that is induced during the macrophage inflammatory response as a common target of a broad range of toll-like receptor (TLR) agonists and inflammatory mediators, such as tumor necrosis factor (TNF) (O’Connell et al. 2007, Chen et al. 2021). *MiR-155* is induced in activated macrophages by direct binding of NF-κB to the *miR-155* gene promoter following TLR activation. *MiR-155* acts as a positive feedback signal to inflammatory responses by repressing the negative regulators SH-2 containing inositol 5’ polyphosphatase 1 (SHIP1) and suppressor of cytokine signaling 1 (SOCS1), or suppressing interleukin (IL)-10 production (Billeter et al. 2014, Doxaki et al. 2015, Mann et al. 2017). *In vivo* data have indicated that *miR-155* promotes lipopolysaccharide (LPS)-induced acute lung injury in mice and rats (Wang et al. 2016) and that *miR-155* overexpression in mice further enhances bacterial infection-induced inflammation (Yang et al. 2021).

Although the role of *miR-155* in inflammatory responses caused by TLR agonists has been intensively studied and well-documented, its effect on host antibacterial defense is less explored. Both beneficial and detrimental effects of *miR-155* on macrophage-mediated antibacterial responses have been reported. *MiR-155* contributes to enhanced phagocytosis of *Streptococcus (S.) pneumoniae* and *Staphylococcus (S.) aureus* by peritoneal macrophages (PMs; Yao et al. 2017), and *miR-155* promotes the bactericidal capacity of the macrophage RAW264.7 cell line against *S. aureus* (Xu et al. 2013). Furthermore, *miR-155* was required for clearance of *S. pneumoniae* colonization in a murine model (Verschoor et al. 2014). Conversely, *miR-155* was documented to interfere with the bactericidal mechanisms in macrophages. For instance, *miR-155* suppressed macrophage-mediated bacterial phagocytosis and intracellular killing of *Pseudomonas aeruginosa* (Yang et al. 2014). Moreover, *miR-155* inhibited apoptosis by destabilizing *Casp-3* mRNA in RAW 264.7 macrophages (De Santis et al. 2016), thereby providing a niche that favors replication of intracellular bacteria, such as *Mycobacterium tuberculosis*, within infected macrophages (Rothchild et al. 2016). Furthermore, *miR-155* increased mortality and impaired bacterial clearance of *S. aureus* in a postviral bacterial pneumonia model (Podsiad et al. 2016).

*Klebsiella (K.) pneumoniae* is a common causative pathogen in hospital-acquired pneumonia and sepsis (Mayr et al. 2014, Rudd et al. 2020). Our previous studies revealed that host defense against *K. pneumoniae* infection is dependent on TLR2 and TLR4 and expression of the TLR adaptor molecule myeloid differentiation factor 88 (MyD88) in myeloid cells (Branger et al. 2004, Wieland et al. 2011, van Lieshout et al. 2014). In view of the importance of *miR-155* in TLR ligand-induced macrophage inflammatory responses, we sought to determine the contribution of myeloid cell specific *miR-155* to the host response during LPS-induced lung inflammation and during pneumosepsis caused by *K. pneumoniae* using mice with a myeloid-specific *miR-155* deficiency and well-established models (de Stoppelaar et al. 2014).

## Methods

### Animals

Homozygous Mir155*^fl/fl^* mice (stocknumber 026700, The Jackson Laboratory) (Hu et al. 2014) were crossed with *LysM^Cre^* mice (Clausen et al. 1999, van Lieshout et al. 2014) to generate myeloid cell specific miR-155-deficient (*Mir155^fl/fl^LysM^Cre^*) mice. *Mir155^fl/fl^* Cre-negative littermates (*Mir155^fl/fl^*mice) were used as controls in all experiments. All genetically modified mice were backcrossed at least eight times to a C57Bl/6 background and age and sex matched when used in experiments. Mice were used at 8–12 weeks of age. All mouse experiments were approved by the Institutional Animal Care and Use Committee of the University of Amsterdam.

### Macrophage preparation

*Bone marrow-derived macrophage (BMDM) generation and stimulation*. Bone marrow cells were cultured in complete medium [CM; RPMI1640 (Gibco) containing 10% FBS, 1% penicillin/streptomycin, 2 mM l-glutamine, and 25 mM HEPES] supplemented with 15% of L929-conditioned medium for 7 days for differentiation (Weischenfeldt and Porse 2008, Qin et al. 2021). BMDMs were then plated at a density of 1 × 10^6^ cells per well in 24-well plates overnight before stimulation with 100 ng/ml ultrapure LPS (*E. coli* O111:B4; InvivoGen), 1 µg/ml Pam3CSK4 (PAM3) (InvivoGen), 1 µg/ml lipoteichoic acid (LTA; InvivoGen), or 20 µg/ml polycytidylic acid (poly(I:C); (InvivoGen). Cell supernatant was collected and stored at −20°C until further analysis and cells were harvested in TRizol (Invitrogen) and stored in −80°C for RNA isolation.

*PM isolation and stimulation*. PMs were harvested by peritoneal lavage and seeded in 48-well flat bottom culture plates (Greiner bio-one) at a density of ∼0.5 × 10^6^ cells per well in CM and left to adhere for 3 hours (Qin et al. 2021). PMs were then washed intensively and stimulated with 100 ng/ml ultrapure LPS for 6 or 24 hours. Cell supernatant was collected and stored at −20°C until further analysis, cells were harvested in TRizol (Invitrogen) and stored in −80°C for RNA isolation.

*Alveolar macrophage (AM) isolation and stimulation*. AMs were harvested by broncho-alveolar lavage (BAL) using a well-established method (Busch et al. 2019) providing pure and functional cells (Otto et al. 2022), and seeded in 96-wells flat bottom culture plates (Greiner bio-one) at a density of ∼5 × 10^4^ cells per well in CM and left to adhere for 3 hours (Qin et al. 2021). AMs were stimulated with 100 ng/ml of ultrapure LPS for 6 hours. Cell supernatant was collected and stored at −20°C until further analysis and cells were harvested in TRizol (Invitrogen) and stored in −80°C for RNA isolation.

### Enzyme-linked immunosorbent assay (ELISA) and cytometric bead array (CBA)

Murine chemokine (C-X-C motif) ligand (CXCL)-1 (KC), CXCL2 (MIP-2), interleukin (IL)-6, TNF, and myeloperoxidase (MPO) in cell culture supernatant and BALF were measured by ELISA’s (R&D Systems) according to manufacturer’s description. IL-6, TNF, and CCL2 (Monocyte Chemotactic Protein-1) in plasma or peritoneal lavage fluid (PLF) were determined using a mouse CBA inflammation kit (BD Biosciences) according to manufacturer’s instructions.

### Quantitative reverse transcription PCR (qRT-PCR)

Total RNA was extracted by using TRizol (Invitrogen) according to the manufacturer’s instruction. To measure miRNA expression, cDNA was synthesized with TaqMan MicroRNA Reverse Transcription Kit (Thermo Fisher). TaqMan miRNA Assays (Applied Biosystems) for mature mmu-mir-155 (Assay ID; 0002571) were performed using a Roche LightCycler 480 thermocycler. Data were analyzed with LinRegPCR based on PCR efficiency values derived from amplification curves (Ramakers et al. 2003). Relative expression of *Mir155* was normalized to U6 snNA (Assay ID; 001973).

### Mouse models

Lung inflammation and pneumonia were induced as previously described (Qin et al. 2022). Briefly, mice were administrated intranasally with 1 µg of ultrapure LPS in 50 µl saline (Otto et al. 2020) and euthanized 6 hours postinoculation. The right lung was used for BAL and the left lung was preserved for histopathology after fixation in 10% formalin. Cell counts in BAL fluid (BALF) were determined using a hemocytometer (Coulter) and different cell populations in BALF samples were determined by flow cytometry (details see below) (de Porto et al. 2019). BALF supernatants were stored at −20°C until further analysis.

Pneumonia was induced by intranasal inoculation of ∼10^4^ CFU *K. pneumoniae* serotype 2 (American Type Culture Collection number 43816). At various time points, BALF and lung tissues were collected in the same way as described for LPS-induced lung inflammation. Blood was collected in heparin minicollect tube (Greiner Bio-One) for direct analysis of bacterial loads and leukocytes or for isolation of plasma and stored at −20°C until further analysis. Spleens and livers were collected and homogenized for bacterial loads or fixed for histopathology. Bacterial loads in blood and tissues homogenates were determined by counting CFU from serial dilutions plated on blood agar plates, incubated at 37°C for 16 hours. Cells in BALF were analyzed as described below. BALF supernatant and plasma were subjected to ELISA or CBA. BALF samples and blood were also collected from naïve mice.

Peritonitis was induced by intraperitoneal injection of 2 mg/kg ultrapure LPS as previously described (Qin et al. 2021) and mice were sacrificed 3 hours later. PLF was isolated for analysis of cells (as described above) or stored at −20°C until further analysis.

### Flow cytometry

Flow cytometry was done on FACS Canto (Becton Dickinson) or CytoFLEX-S (Beckman Coulter) and data were analyzed using FlowJo software (Becton Dickinson) as described (de Porto et al. 2019, Qin et al. 2020). BALF and PLF cells were resuspended in FACS buffer (0.5% BSA, 0.35 mM EDTA, 0.01% NaN3) and stained according to manufacturer’s recommendations using fixable viability dye eFluor 780, rat antimouse CD16/CD32 (clone 93), rat antimouse-CD45 PE-eFluor610 (clone 30-F11), rat antimouse CD11b PE-Cy7 (clone M1/70), rat antimouse Siglec-F Alexa Fluor 647 (clone E50-2440), rat antimouse Ly-6C Alexa Fluor700 (clone AL-21), rat antimouse F4/80 APC (BM8; PLF cells only) (all from BD Biosciences); and rat antimouse Ly-6 G FITC (clone 1A8; Biolegend). The gating strategy for BALF and PLF leukocytes was performed as previously (Qin et al. 2021).

### Histology

Paraffin embedded lungs and livers were cut into 4-µm sections and stained with hematoxylin and eosin (H&E). Lung inflammation was scored by a pathologist blinded for treatment and genotype of the mice as previously described (Meijer et al. 2021) for the following parameters: bronchitis, edema, interstitial inflammation, intra-alveolar inflammation, pleuritis, endothelialitis, and percentage of the lung surface demonstrating confluent inflammatory infiltrate. Each parameter was graded 0–4, with 0 being “absent” and 4 being “severe;” the total pathology score was expressed as the sum of the score for all parameters with a maximum score of 32. Liver pathology was scored as previously described (Claushuis et al. 2018) for inflammation, necrosis, and presence of thrombi, with a maximum score of 12.

### Clinical chemistry

Aspartate aminotransferase (AST), alanine aminotransferase (ALT), and lactate dehydrogenase (LDH) in plasma were measured using a c702 Roche Diagnostics analyzer (Roche Diagnostics, Almere, the Netherlands).

### Statistical analysis

Nonparametric variables were analyzed using the Mann–Whitney U-test. Analysis was done using GraphPad Prism version 8 (Graphpad Software, San Diego, CA). Statistical significance is shown as * *P* < .05; ** *P* < .01.

## Results

### Myeloid *miR-155* deficiency does not affect immune cell development in mice

While *miR-155* is known to be involved in the regulation of inflammation during infection and sepsis (Chen et al. 2021), the role of *miR-155* in myeloid cells, the major class of immune effector cells during infection, is less well-explored. Thus, we here sought to determine the role of myeloid *miR-155 in vitro* and *in vivo* during inflammation induced by the Gram-negative bacterial cell wall component LPS or by *K. pneumoniae* infection. To this end, we generated myeloid cell specific *miR-155-*deficient mice (*Mir155^fl/fl^LysM^Cre^*) and littermate controls (*Mir155^fl/fl^*). In AMs, PMs and BMDMs of *Mir155^fl/fl^LysM^Cre^* mice, *miR-155* expression was decreased by 72%, 80%, and 65%, respectively, compared to cells of *Mir155^fl/fl^*littermates (Figure S1A, Supporting Information). As previous studies revealed that *miR-155* deficiency affects the development of immune cells (Kohlhaas et al. 2009, Mann et al. 2017, Pashangzadeh et al. 2021), we assessed whether deficiency of *miR-155* in myeloid cells impacted on myeloid populations in the alveolar lumen of the lung and in blood. AMs are the dominant cell population in the lung lumen, while neutrophils and monocytes numbers are very low. No differences were found in these cell populations between naïve *Mir155^fl/fl^LysM^Cre^* mice and littermate controls (Figure S1B, Supporting Information). Likewise, the myeloid cell populations in blood were also not affected by *miR-155* deficiency in myeloid cells (Figure S1C, Supporting Information). Taken together, these data indicate that *miR-155* deficiency in the myeloid lineage does not affect myeloid cell numbers in the lung and circulation.

### Myeloid *miR-155* deficiency represses TLR ligands-induced macrophages activation *in vitro*

**Figure 1.**
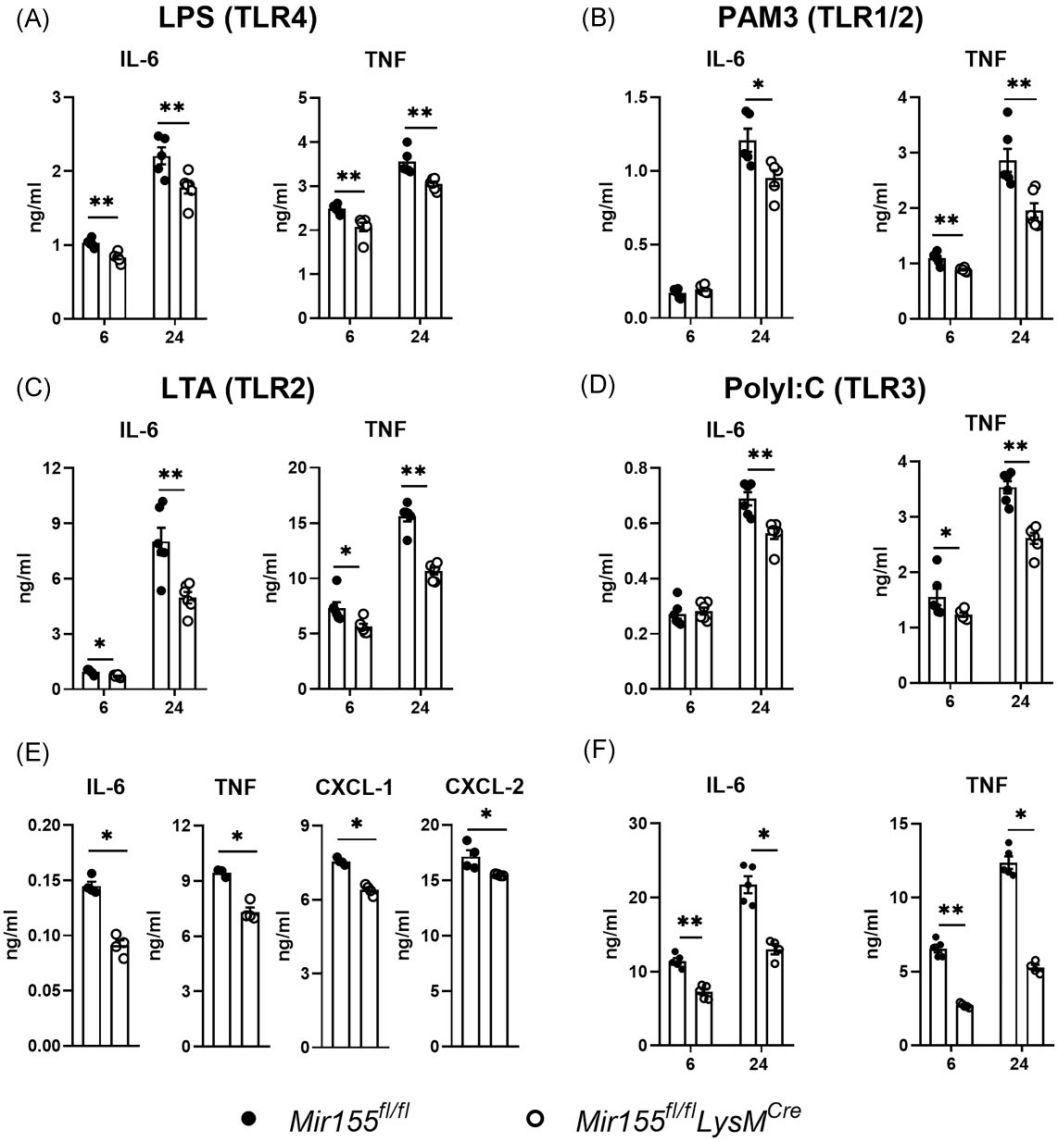
*MiR-155* augments cytokine secretion by TLR ligand-stimulated macrophages *in vitro*. (A)–(D) BMDMs from myeloid specific *miR-155-*deficient mice (*Mir155^fl/fl^LysM^Cre^*) or littermate control mice (*Mir155^fl/fl^*) were stimulated with LPS, PAM3CSK4, LTA and poly(I:C), respectively. After 6–24 hours, IL-6 and TNF levels were determined by ELISA; (E) AMs isolated from myeloid specific *miR-155* mice (*Mir155^fl/fl^LysM^Cre^*) or littermate control mice (*Mir155^fl/fl^*) were stimulated with LPS for 6 hours; IL-6, TNF, CXCL-1, and CXCL-2 levels were determined by ELISA; (F) PMs were isolated from *Mir155^fl/fl^LysM^Cre^* or littermate control mice and stimulated with LPS for 6 or 24 hours; IL-6, and TNF levels were determined by ELISA. *N* = 4. Data is shown as bar graphs with mean ± SD with individual values. *P*-values were calculated using Mann–Whitney test. * *P* < .05, ** *P* < .01.

Since *miR-155* is known to regulate inflammatory responses during infections (Chen et al. 2021), we investigated the role of *miR-155* in cytokine secretion by macrophages *in vitro* in response to various TLR ligands. To this end, we generated BMDMs and isolated primary AMs and PMs of *Mir155^fl/fl^LysM^Cre^* and *Mir155^fl/fl^*control mice and stimulated these with LPS (TLR4 ligand), PAM3CSK4 (TLR1/2 ligand), LTA (TLR2 ligand), or poly(I:C) (TLR3 ligand). In *miR-155-*deficient BMDMs, secretion of both IL-6 and TNF was decreased at either 6 or 24 hours after stimulation with all these TLR ligands, as compared to wild type BMDMs (Fig. 1A–D). Compared to wild type AMs, *miR-155-*deficient AMs released significantly less proinflammatory cytokines IL-6 and TNF, and chemokines CXCL1 and CXCL2 in response to LPS (Fig. 1E). Likewise, PMs of *Mir155^fl/fl^LysM^Cre^* mice secreted less IL-6 and TNF after stimulation with LPS when compared to PMs from the *Mir155^fl/fl^*control mice (Fig. 1F). These results indicate that myeloid specific deficiency of *miR-155* attenuates inflammatory responses in macrophages upon activation by TLR ligands *in vitro*, corroborating previous findings from *in vitro* experiments with cells from constitutive *miR-155-*deficient mice (Taganov et al. 2006, Mann et al. 2017).

### Myeloid *miR-155* deficiency does not affect LPS-induced inflammation *in vivo*

**Figure 2.**
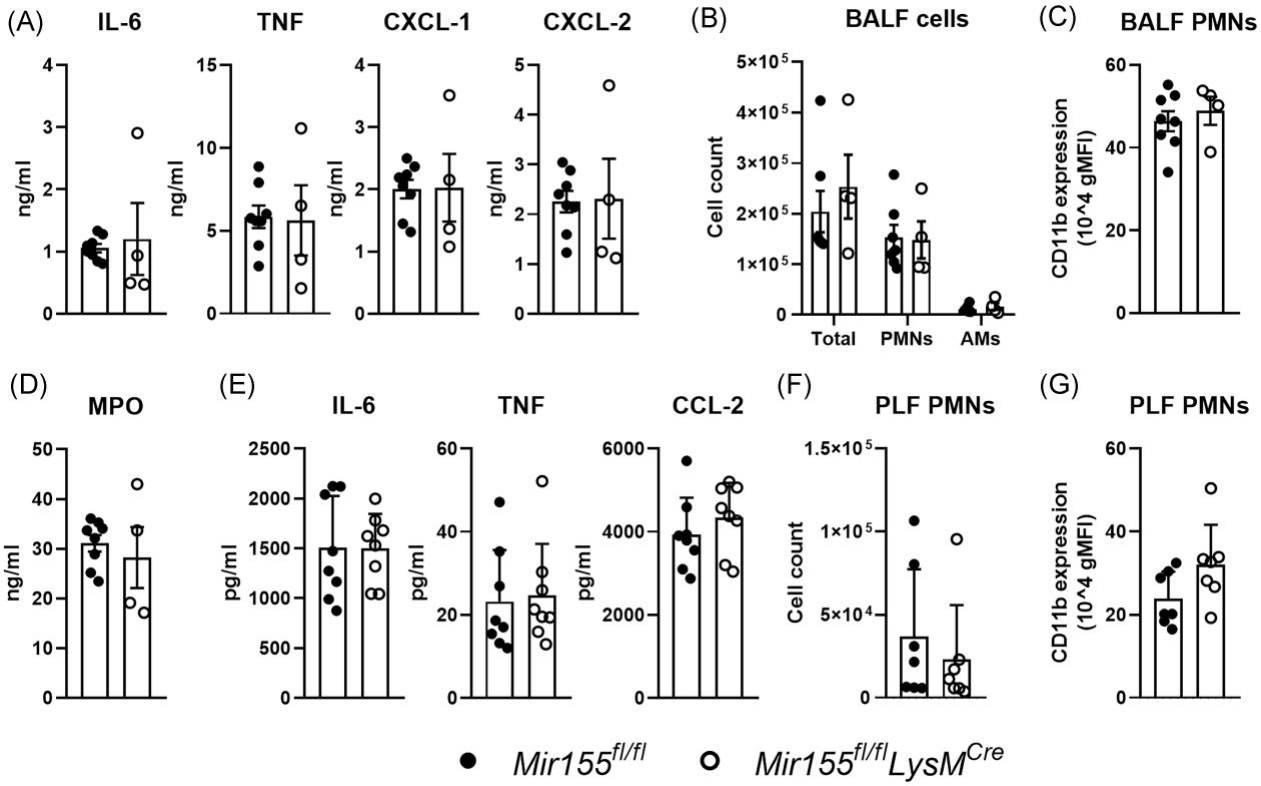
Myeloid *miR-155* deficiency does not affect LPS-induced lung and peritoneal inflammation *in vivo*. (A) IL-6, TNF, CXCL-1, and CXCL-2 levels, (B) number of total leukocytes, neutrophils (PMNs), and AMs, (C) neutrophil CD11b expression and (D) MPO levels in BALF from myeloid *miR-155-*deficient mice (*Mir155^fl/fl^LysM^Cre^*) and control mice (*Mir155^fl/fl^*) 6 hours after administration of LPS (1 µg/mouse) via the airways. *N* = 8 for *Mir155^fl/fl^* and *n* = 4 for *Mir155^fl/fl^LysM^Cre^* mice. (E) IL-6, TNF, and CCL-2 protein levels, (F) number of neutrophils, and (G) neutrophil CD11b expression in PLF from *Mir155^fl/fl^LysM^Cre^* and *Mir155^fl/fl^* control mice 3 hours after intraperitoneal administration of LPS (2 mg/kg). *N* = 8 for both *Mir155^fl/fl^* and *Mir155^fl/fl^LysM^Cre^* mice. Data are shown as bar graphs with mean ± SD and individual values. Data between *Mir155^fl/fl^LysM^Cre^* and control *Mir155^fl/fl^* mice were not significantly different.

To determine the role of myeloid *miR-155* in lung inflammation *in vivo*, we first investigated the inflammatory response in the lung of mice challenged with LPS via the airways. Analysis of BALF for hallmark inflammatory reactions revealed that, opposite to our *in vitro* results, myeloid specific *miR-155*-deficient mice displayed comparable levels of IL-6, TNF, CXCL1, and CXCL2 during LPS-induced lung inflammation (Fig. 2A). Furthermore, leukocyte numbers and neutrophil influx (Fig. 2B), as well as neutrophil activation (as indicated by CD11b expression and MPO levels in BALF) (Fig. 2C and D) were also not affected by *miR-155* deficiency in myeloid cells. In view of the robust expression of *miR-155* in PMs (Figure S1A, Supporting Information), we also investigated the role of myeloid *miR-155* in the inflammatory response in the peritoneal cavity after intraperitoneal injection of LPS. Similar to LPS-induced lung inflammation, LPS-induced peritonitis in myeloid *miR-155-*deficient mice was not associated with an altered cytokine and chemokine response (Fig. 2E); Similarly, the neutrophil influx (Fig. 2F), as well as neutrophil activation (as indicated by CD11b expression, Fig. 2G) were also comparable between myeloid miR-155-deficient mice and littermate controls. Taken together these results indicate that *miR-155* in myeloid cells does not impact on innate immune responses during acute LPS-induced inflammation *in vivo*.

### Myeloid *miR-155* deficiency does not impact on host defense and inflammatory responses in the lung during *K*. pneumonia

**Figure 3.**
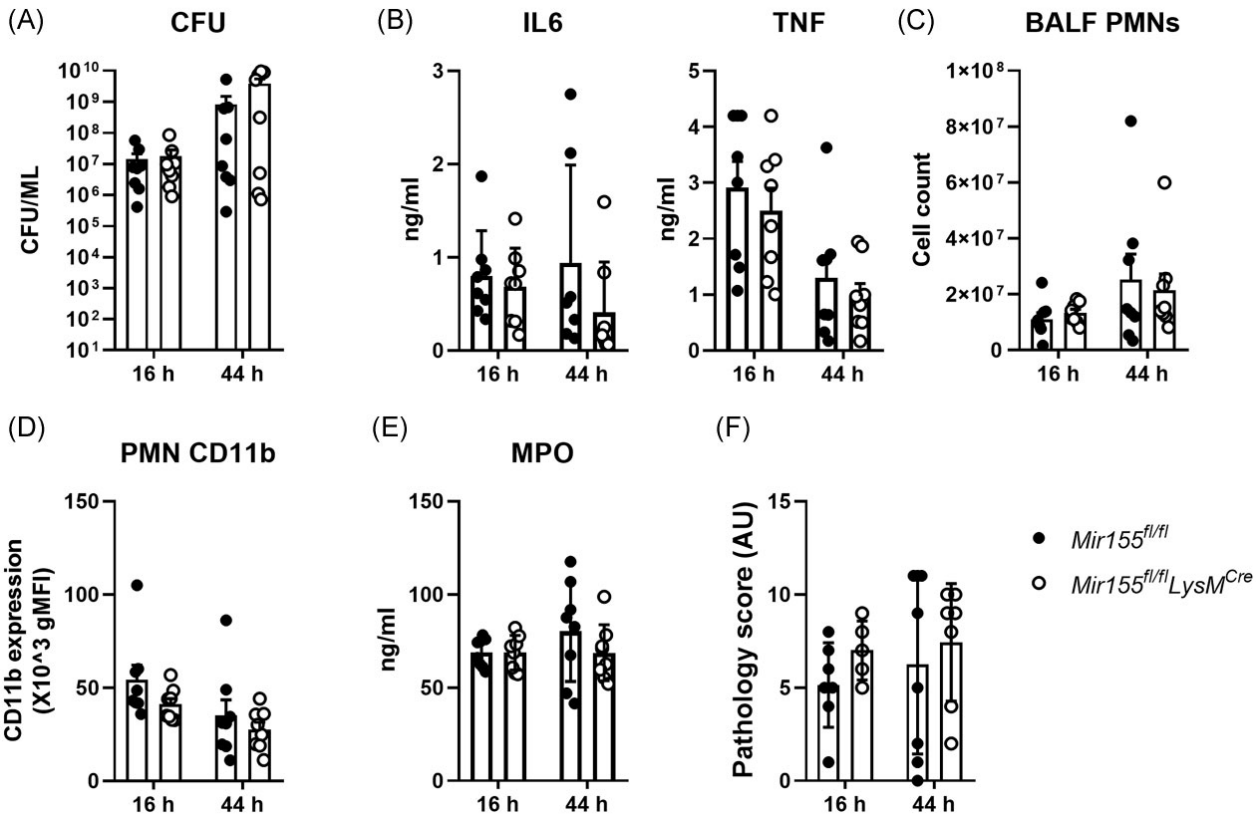
Myeloid *miR-155* deficiency does not impact on host defense and inflammatory responses in the lung during *K*. pneumonia. Myeloid miR-155-deficient mice (*Mir155^fl/fl^LysM^Cre^*) and control mice (*Mir155^fl/fl^*) were infected with 10^4^* K. pneumoniae* via the airways for 16 or 44 hours. (A) Bacterial burden (CFU) in lung, (B) IL-6 and TNF levels, (C) neutrophil influx (PMNs), and (D) neutrophil CD11b expression and MPO levels in BALF. (E) Lung pathology scored according to the semiquantitative scoring system described in the Methods. *N* = 8. Data are shown as bar graphs with mean ± SD and individual values. Data between *Mir155^fl/fl^LysM^Cre^* and control *Mir155^fl/fl^* mice were not significantly different.

Previous studies have shown that *miR-155* critically regulates host defense against bacterial infection (Mashima 2015, Mann et al. 2017, Chen et al. 2021). TLR2 and TLR4 signaling are required for host defense against *K. pneumoniae* (Branger et al. 2004, Wieland et al. 2011), and since miR-155 is involved in macrophage inflammatory responses evoked by TLR2 and TLR4 agonists (Fig. 1), we decided to determine the effect of myeloid *miR-155* deficiency on the pulmonary immune response during *K. pneumoniae* infection, using a well-studied model evoked by intranasal inoculation of 10^4^ CFU of hypervirulent *K. pneumoniae* (de Stoppelaar et al. 2014). Bacterial loads in lung tissue at 16- or 44-hours postinfection were not affected by myeloid *miR-155* deficiency (Fig. 3A). Lung inflammation was then evaluated in *Mir155^fl/fl^LysM^Cre^* and *Mir155^fl/fl^* control mice by measuring IL-6 and TNF levels, neutrophil influx and neutrophil activation (CD11b expression and MPO levels) in BALF. Our data showed that none of these measures was affected at both time points by *miR-155* deficiency in myeloid cells (Fig. 3B–E). Moreover, analysis of lung pathology also did not reveal impact of myeloid miR-155 deficiency on *Klebsiella*-induced lung inflammation (Fig. 3F). These results indicate that myeloid miR-155 is dispensable for pulmonary host defense and inflammation during *Klebsiella* pneumonia.

### Myeloid *miR-155* deficiency impacts host defense in the liver but does not affect systemic inflammatory responses during *Klebsiella* pneumosepsis

**Figure 4.**
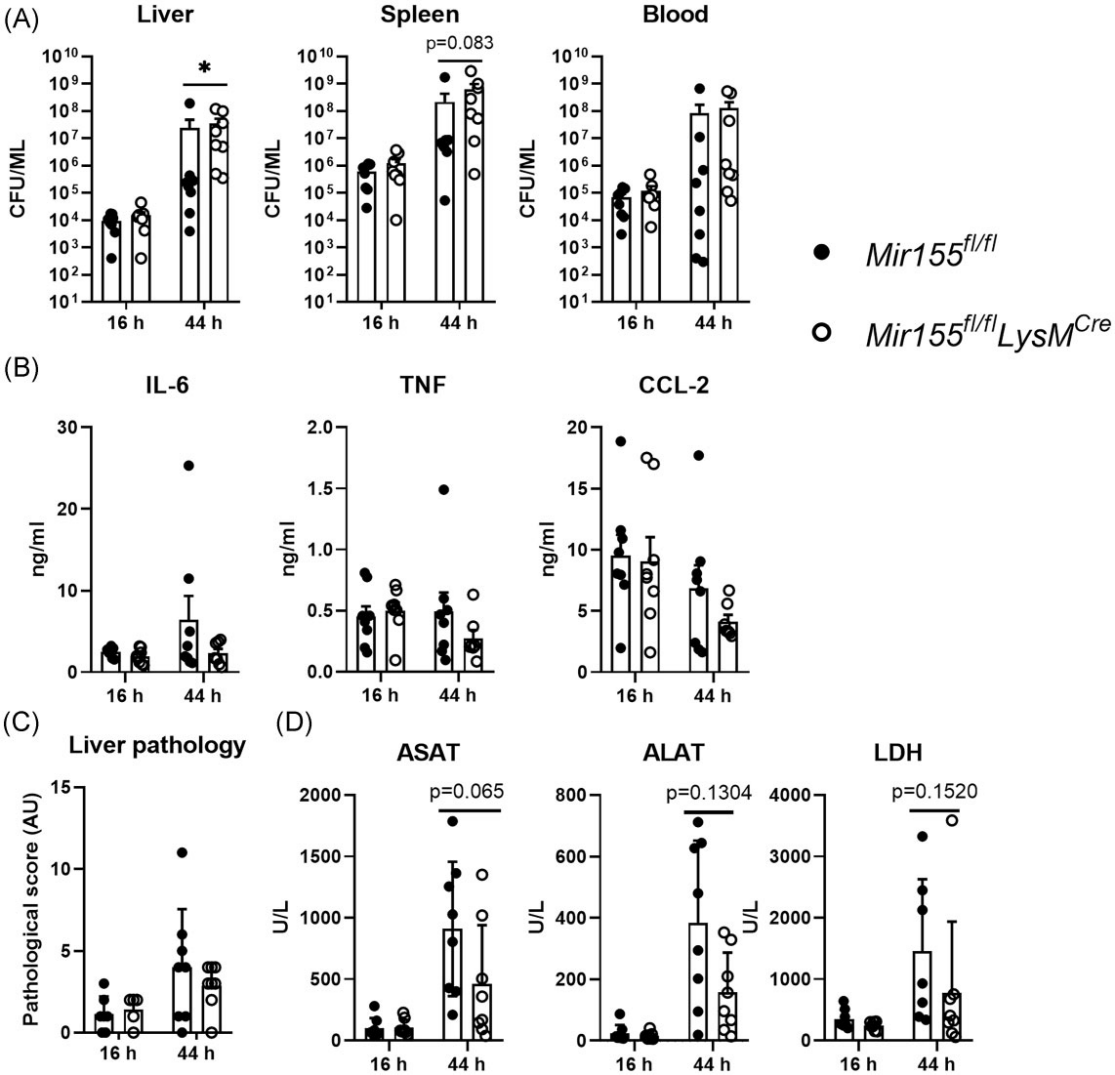
Myeloid *miR-155* deficiency impacts host defense in the liver and bacterial dissemination but does not affect systemic inflammatory responses during *Klebsiella* pneumosepsis. *Mir155^fl/fl^LysM^Cre^* and control *Mir155^fl/fl^* mice were infected with 10^4^* K. pneumoniae* via the airways for 16 or 44 hours. (A) Bacterial burden (CFU) in liver, spleen, and blood. (B) IL-6, TNF, and CCL-2 protein levels in plasma. (C) Liver pathology scored according to the semiquantitative scoring system described in the section “Methods.” (D) Organ damage parameters AST, ALT, and LDH levels in plasma. *N* = 8. Data are shown as bar graphs with mean ± SD with individual values. *P-*values were calculated using Mann–Whitney test. * *P* < .05.

Since the *K. pneumoniae* strain used in our study can disseminate and cause systemic inflammation and organ damage (Claushuis et al. 2016), we next analyzed the bacterial burden in blood and distant organs (spleen and liver) at 16 or 44 hours postinfection. Bacterial loads in the liver of *Mir155^fl/fl^LysM^Cre^* mice were significantly higher than in littermate controls at 44 hours after infection, while in blood and spleen a similar trend was observed (Fig. 4A). To gain insight into the effect of myeloid *miR-155* on systemic inflammatory responses, we measured levels of IL-6, TNF, and CCL2 levels in plasma from *Mir155^fl/fl^LysM^Cre^* and control mice. None of the mediators were affected by myeloid miR-155 deficiency (Fig. 4B). Likewise, neither neutrophil numbers nor neutrophil activation (CD11b expression) in blood were altered by *miR-155* deficiency in myeloid cells (Figure S2, Supporting Information). In view of the higher bacterial loads in the liver of *Mir155^fl/fl^LysM^Cre^* mice, we examined liver pathology and plasma levels of AST, ALT (reflecting hepatocellular injury). Histopathological changes in liver were equal between *Mir155^fl/fl^LysM^Cre^* and *Mir155^fl/fl^* mice (Fig. 4C). Measurement of plasma AST and ALT, as well as LDH (indicative of cellular injury in general) showed that these parameters were increased at 44 hours postinfection and were slightly but not significantly lower in myeloid *miR-155-*deficient mice at this time point (Fig. 4D). Together these data suggest that myeloid *miR-155* contributes to host defense in the liver but may promote liver damage while being dispensable for regulation of systemic inflammatory responses during *K. pneumoniae*-derived pneumosepsis.

## Discussion

*MiR-155* is an important regulatory molecule for inflammatory responses in multiple cell types. We here investigated its function in myeloid cells, crucial components of the immune response during bacterial infection, both *in vitro* and *in vivo*. Our data indicates that *miR-155* potentiates inflammatory responses of macrophages in an *in vitro* setting, with reduced cytokine secretion by *miR-155*-deficient macrophages after stimulation with multiple TLR ligands, while myeloid *miR-155* deficiency had no effect on inflammatory responses *in vivo* during LPS-induced inflammation or intact *K. pneumoniae* infection. Interestingly, we found that *miR-155* might play a role in host defense in the liver during *Klebsiella* pneumosepsis.

Overall genetic depletion of *miR-155* leads to abnormalities in the development of the immune system (Kohlhaas et al. 2009, Mann et al. 2017, Pashangzadeh et al. 2021). Unlike most previous studies with constitutive *miR-155*-deficient mice, we here used myeloid cell specific *miR-155-*deficient mice that are phenotypically normal and showed an unaltered immune cell development. Our *in vitro* data showed that *miR-155* potentiates TLR ligand-induced inflammatory mediator production in macrophages, which is in agreement with results from previous studies using mice with constitutive deficiency of *miR-155* (Mann et al. 2017). Strikingly, we found that, opposite to our findings with AMs and PMs *in vitro*, wherein *miR-155* deficiency was associated with reduced IL-6, TNF, CXCL-1, and CXCL-2 production, upon *in vivo* targeting of the sites (lung or peritoneal cavity) where these tissue macrophages reside *miR-155* deficiency was not associated with lowered cytokine responses. The most likely explanation for the discrepancy between these *in vivo* and *in vitro* data is the contribution of different cell types besides macrophages to the production of these mediators in animals. Additionally, miR-155 can be produced and secreted by other nonimmune cells (Zheng et al. 2017, Jiang et al. 2019), thus miR155 signalling is likely to be functionally maintained in macrophages of *Mir155^fl/fl^LysM^Cre^* mice *in vivo*. Hence, although *miR-155* potentiates the production of various cytokines in tissue macrophages from different organs, its deficiency in macrophages in mice *in vivo* seems to have a lesser effect on host inflammatory responses.

Myeloid cells, via TLR-dependent signaling, play an important role in antibacterial defense during *K. pneumoniae* infection (Branger et al. 2004, Wieland et al. 2011, van Lieshout et al. 2014). Previous studies have indicated that TLR activation induces the expression of *miR-155* expression in macrophages (O’Connell et al. 2007). In agreement with this, *miR-155* expression was increased in the murine AM cell line RAW264.7 after infected with *K. pneumoniae* (Teng et al. 2016). Furthermore, *miR-155* was suggested to augment phagocytosis of *S. pneumoniae* and *S. aureuvs* by macrophages in a TLR2-dependent manner (Yao et al. 2017). Additionally, *miR-155* promotes neutrophil extracellular trap formation, which is known as a crucial mechanism for controlling *K. pneumoniae* infection in mice (Claushuis et al. 2018, Hawez et al. 2022). All these mechanisms may explain our finding that the bacterial burden in myeloid *miR-155*-deficient mice was higher in liver than in littermate control mice. Recently, it was reported that Kupffer cells in the liver, the largest population of tissue resident macrophages, can sequester hypervirulent *Klebsiella* intracellularly and provide a niche for bacterial persistence (Wanford et al. 2021). Further investigation, however, is required to determine the mechanism by which *miR-155* mediates antibacterial defense against *Klebsiella* in the liver and whether *miR-155* in Kupffer cells is involved in phagocytosis, killing or sheltering of *Klebsiella*.

Pneumonia is the leading cause of sepsis (Torres et al. 2021), which is defined as a life-threatening organ dysfunction caused by a dysregulated host response to an infection (Singer et al. 2016). Emerging evidence from both clinical investigations and murine experiments has revealed that *miR-155* is involved in organ damage during sepsis (Liu et al. 2015, Hawez et al. 2022). *MiR-155* was significantly upregulated in blood leukocytes of sepsis patients (Liu et al. 2015). Plasma *miR-155* levels were elevated in patients with septic cardiac dysfunction (Wang et al. 2016) and indicative of a more severe condition and poorer prognosis (Liu et al. 2015). Therefore, a high level of *miR-155* was suggested as a potential biomarker for predicting mortality and treatment outcome of severe sepsis in patients (Liu et al. 2015, Han et al. 2016). The same phenotype was captured in mouse sepsis models. *MiR-155* expression was significantly higher in septic mice and associated with accelerated lung and liver injury (Tuerdi et al. 2018, Yang et al. 2018), while the inhibition of *miR-155* attenuated sepsis-induced liver and lung damage and increased the survival rate of septic mice (Lv et al. 2015, Tuerdi et al. 2018, Yang et al. 2018). In the present study, we found that organ damage parameters (LDH, ALT, and AST) showed a tendency for lower levels in myeloid *miR-155-*deficient mice after infection with *K. pneumoniae*, suggesting that *miR-155* in myeloid cells is sufficient to augment sepsis-induced organ damage. These findings, however, were not corroborated by significant histopathological changes in the liver (or lung). A possible reason for this discrepancy might be that the *K. pneumoniae* infection causes mild histopathological changes in the lung and liver, as shown by low pathological scores in both groups even at 44 hours after infection, that are not sufficient to distinguish a role of *miR-155* by this method.

As a limitation of our study, it is worth noting that the bacterial strain used in this study is a mouse-adapted strain, and the host responses may vary significantly with different bacterial strains (Bengoechea and Sa Pessoa 2019). Moreover, the outcome of the infection can be notably influenced by specific strains of *Klebsiella* (Wanford et al. 2021). Therefore, we cannot rule out that *miR-155* deficiency in myeloid cells might have a different impact on the host’s immune response when encountered by another *Klebsiella* strain. Further investigation is warranted to understand the impact of *miR-155* deficiency on the host immune responses against various strains of *Klebsiella*. Further studies with purified macrophages from affected organs are also required to elucidate the apparent discrepancy between *in vitro* and *in vivo* cytokine results, since infiltrating cells and parenchymal cells may contribute to cytokine levels *in vivo*. Another limitation of our study is that the levels of *miR-155* in macrophages of *Mir155^fl/fl^LysM^Cre^* mice did not result in complete reduction, which could explain the limited effect of the *miR-155* deficiency. Previously, we have shown that *LysM^Cre^* largely, but not completely reduced protein levels of the targeted genes in AMs, PMs, and BMDMs (Anas et al. 2016, de Porto et al. 2021, Otto et al. 2022). In the current study, we used a TaqMan miRNA assay to analyze the expression of the target gene, which may be more sensitive for residual expression in *LysM^Cre^* targeted cells. Another explanation for *miR-155* in these cells may be uptake of *miR-155* carrying microvesicles derived from other cells (Gomez et al. 2020).

In conclusion, we here used *Mir155^fl/fl^LysM^Cre^* mice to assess the role of myeloid cell-specific *miR-155* and show that *miR-155* acts as a potentiator of proinflammatory cytokine and chemokine release by various types of macrophages upon exposure to various TLR ligands. The impact of myeloid cell *miR-155* deficiency on inflammatory responses during LPS-induced lung and peritoneal inflammation and during *K. pneumoniae*-evoked pneumosepsis was limited. Interestingly, our data suggest that the myeloid cell *miR-155* may play a role in the regulation of host antibacterial defense against *Klebsiella* in the liver.

## Supplementary Material

ftad031_Supplemental_File
