# Supplementary material for: Myeloid miR-155 plays a limited role in antibacterial defense during Klebsiella-derived pneumosepsis and is dispensable for lipopolysaccharide- or Klebsiella-induced inflammation in mice
Source: Pathog Dis. 2023 Oct 19;81:ftad031. doi: 10.1093/femspd/ftad031 (PMC10636497; doi:10.1093/femspd/ftad031)
Supplement: ftad031_Supplemental_File [file ftad031_supplemental_file.docx]

**Supplementary materials**

Myeloid *miR-155* plays a limited role in antibacterial defense during *Klebsiella*-derived pneumosepsis and is dispensable for lipopolysaccharide- or *Klebsiella*-induced inflammation in mice

Wanhai Qin ^1,2^, Anno Saris ^1,2^, Cornelis van ’t Veer ^1,2^, [Joris J T H Roelofs](https://pubmed.ncbi.nlm.nih.gov/?sort=date&term=Roelofs+JJTH&cauthor_id=35011643) ^3,4^,  Brendon P. Scicluna ^1,2,5,6^, Alex F. de Vos ^1,2^, Tom van der Poll ^1,2,7^

1. Center for Experimental and Molecular Medicine, Amsterdam University Medical Centers, Academic Medical Center, University of Amsterdam, Amsterdam, the Netherlands.
2. Amsterdam Infection & Immunity Institute, Amsterdam, The Netherlands.
3. Department of Pathology, Amsterdam University Medical Centers, Academic Medical Center, University of Amsterdam, Amsterdam, the Netherlands.
4. Amsterdam Cardiovascular Sciences, University of Amsterdam, Amsterdam, the Netherlands
5. Department of Applied Biomedical Science, Faculty of Health Sciences, Mater Dei Hospital, University of Malta, Msida, Malta.
6. Centre for Molecular Medicine and Biobanking, University of Malta, Msida, Malta.
7. Division of Infectious Diseases, Amsterdam University Medical Centers, University of Amsterdam, Amsterdam, the Netherlands.

Corresponding author:

Wanhai Qin

[wanhai.qin1@ucalgary.ca](mailto:wanhai.qin1@ucalgary.ca)

**Supplementary Figure 1. Myeloid specific *miR-155* deficiency does not affect immune cell development. (A**) . *MiR-155* expression in alveolar macrophages (AMs), peritoneal macrophages (PMs) and bone marrow-derived macrophages (BMDMs) from myeloid specific *miR-155* deficient mice (*Mir155^fl/fl^LysM^Cre^*) or littermate control mice (*Mir155^fl/fl^*) was measured by qPCR*.* **(B, C)** Myeloid cell populations (Alveolar macrophage, neutrophils, inflammatory monocytes, and non-inflammatory monocytes) in bronchoalveolar lavage fluid (BALF) and myeloid cells (Neutrophils, inflammatory monocytes, and non-inflammatory monocytes) in blood of naïve *Mir155^fl/fl^LysM^Cre^* and *Mir155^fl/fl^* littermate control mice. N = 4. Data are shown as bar graphs with mean ± SD with individual values.

**Supplementary Figure 2. Myeloid *miR-155* deficiency does not affect cell composition in the blood during *Klebsiella*-evoked pneumosepsis.** *Mir155^fl/fl^LysM^Cre^* and *Mir155^fl/fl^* littermate control mice were infected with *K. pneumoniae* via the airways and blood and liver tissue was collected 16 or 44 hours later. **(A)** PMN percentage in blood and **(B)** CD11b expression on blood neutrophils. N = 8. Data are shown as bar graphs with mean ± SD with individual values.
